# Naringenin inhibits ferroptosis to reduce radiation-induced lung injury: insights from network Pharmacology and molecular docking

**DOI:** 10.1080/13880209.2025.2465312

**Published:** 2025-02-19

**Authors:** Junlin Jiang, Xianhui Deng, Chengkai Xu, Yaxian Wu, Jianfeng Huang

**Affiliations:** a Department of Radiation Oncology, Affiliated Hospital of Jiangnan University, Wuxi, China; b Department of Neonatology, Jiangyin People’s Hospital of Nantong University, Wuxi, China; c Wuxi School of Medicine, Jiangnan University, Wuxi, China

**Keywords:** Naringenin, irradiation, ferroptosis, RILI, network pharmacology, Molecular docking

## Abstract

**Context:**

Naringenin is a natural flavanone with potent pharmacological properties. It has demonstrated therapeutic potential in treating various diseases and organ injuries, including radiation-induced lung injury (RILI). Ferroptosis is a newly type of cell death, and naringenin has been shown to attenuates ferroptosis.

**Objective:**

To evaluate the inhibitory effect and molecular mechanism of naringenin on ferroptosis during RILI process.

**Materials & methods:**

Firstly, BEAS-2B and HUVECs cells were pre-incubated with naringenin for 1 h prior to 8 Gy of X-ray irradiation to evaluate oxidative stress, inflammation, and the mRNA levels of ferroptosis-related genes. Next, target genes of naringenin, RILI, and ferroptosis were identified using the TCMSP, SwissTargetPrediction, and GeneCards databases. The target network was constructed with Cytoscape and STRING. Finally, the core target genes were identified through *in vitro* experiments by qRT-PCR, western blot and immunofluorescence staining.

**Results:**

Naringenin effectively reduced radiation-induced increasement of oxidative stress, inflammation, and ferroptosis markers in both cell lines. Network pharmacology identified 14 target genes, with prostaglandin endoperoxide synthase (PTGS2) and Valosin-containing protein (VCP) mRNA levels being prominent, which were crucial for ferroptosis regulation. Molecular docking revealed strong binding interactions between naringenin and the two target proteins. Subsequently, experimental validation confirmed that naringenin reduced the elevated levels of PTGS2 and VCP induced by radiation.

**Discussion & conclusion:**

Naringenin alleviates radiation-induced lung damage by inhibiting ferroptosis, with PTGS2 and VCP emerging as potential therapeutic targets.

## Introduction

Radiation-induced lung injury (RILI) is a common complication in patients undergoing thoracic radiotherapy, affecting nearly 50% of patient post-treatment (Wang X, Li M, et al. 2023). RILI manifests as inflammation, lung deterioration, fibrosis, progressive dyspnea, and ultimately respiratory failure, posing a serious threat to patients’ lives (Arroyo-Hernández et al. 2021). At present, there is still no effective treatment for RILI. Hormones are commonly used in clinical settings, but the curative effect is poor, and the side effects are significant.(Oray et al. 2016; Bledsoe et al. 2017). Therefore, it is urgent to explore and develop new radioprotective drugs that can effectively prevent and treat RILI, with low toxicity and minimal side effects for clinical management.

The pathogenesis of RILI remains incompletely understood, with numerous cell types and cytokines playing crucial roles in its occurrence and progression (Yan et al. 2022; Guo et al. 2024). Ferroptosis represents a novel form of cell death characterized by iron accumulation, lipid per oxidation, and the production of reactive oxygen species (ROS) (Macías-Rodríguez et al. 2020; Zhang et al. 2024). Prostaglandin endoperoxide synthase (PTGS2) has been reported as a key gene regulating ferroptosis in cervical cancer and atherosclerosis (Zhou et al. 2021; Zou et al. 2022). Recent studies have found that ferroptosis plays a key role during RILI (Zhang et al. 2024). The accumulation of ROS is the primary factor that triggers ferroptosis in RILI (Li et al. 2019). Furthermore, irradiation can induce ferroptosis in RILI by inhibiting GPX4 and reducing GSH levels (Ning et al. 2024). Therefore, it is urgent to search for new mechanisms and drugs to target or modulate ferroptosis in RILI.

Naringenin, classified as a flavanone, is a phytochemical compound exhibiting significant biological activity and predominantly found in citrus fruits (Motallebi et al. 2022). Due to its powerful antioxidant capacity to reduce tissue damage and fibrosis (Maleki et al. 2019), some studies have shown that naringenin can improve radiation-induced intestinal, lung, and submandibular gland injury (Zhang et al. 2018; Sakat et al. 2022; Ling et al. 2024). Naringenin protects cells by inhibiting radicals, neutralizing ROS/RNS, and reducing pro-inflammatory cytokines, which also play crucial roles in the process of ferroptosis (Chen GL et al. 2019; Chen X et al. 2021). Studies have found that naringenin could reduce ferroptosis in inflammatory diseases. Zhang et al. report that naringenin could activate Nrf2/HO-1 pathway, thereby alleviating lung injury induced by silver nanoparticles through the inhibition of ferroptosis (Zhang et al. 2022). Additionally, Xu et al. and Hou et al. demonstrate that naringenin inhibits ferroptosis by regulating Nrf2/SystemXc-/GPX4 and targeting YAP/STAT3, resulting in the alleviation of myocardial and intestinal ischemia/reperfusion injury, respectively (Xu et al. 2021; Hou et al. 2024). However, whether naringenin alleviates RILI *via* ferroptosis and the regulatory mechanism remain unclear.

**Figure 1.**
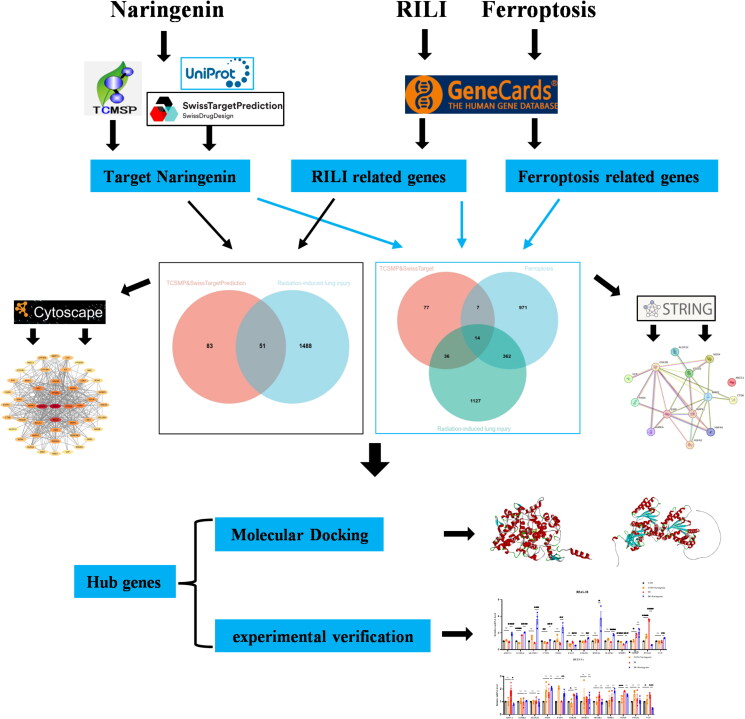
Experimental flow chart. This figure illustrates the working process of the study. Initially, the target genes of naringenin were acquired through screening the TCMSP and SwissTargetPrediction databases. Subsequently, RILI-related genes and ferroptosis-related genes were obtained from the GeneCards database. Then, the naringenin -related and RILI -related gene data were intersected to identify 51 common genes. The three sets of genetic data were intersected to obtain 14 common genes. The construction of a protein–protein interaction (PPI) network by String and Cytoscape was performed to identify hub genes. The candidate hub genes were then subjected to molecular docking and experimental verification.

The main purpose of this study was to explore the targets of naringenin associated with ferroptosis in RILI using network pharmacology and molecular docking techniques (Figure 1). The results revealed that PTGS2 and Valosin-containing protein (VCP) were key targets of naringenin regulating ferroptosis hence alleviating RILI. This discovery provides new insights into the potential therapeutic targets for RILI.

## Materials and methods

### Naringenin pharmacokinetic parameter analysis

The pharmacokinetic parameters of naringenin were obtained from the Traditional Chinese Medicine Systems Pharmacology Database and Analysis Platform (TCMSP) database.

### Naringenin target gene acquisition

Naringenin target genes were identified using the Traditional Chinese Medicine Systems Pharmacology Database and Analysis Platform (TCMSP) and the SwissTargetPrediction platforms (Ru et al. 2014; Zhang et al. 2021). These target genes were then cross-referenced with the Universal Protein (UniProt) database (www.uniprot.org) to confirm their exact nomenclature. The target genes identified by both platforms were subsequently combined to establish the predicted target genes of naringenin.

### RILI and ferroptosis related genes acquisition

‘RILI’ was used as a keyword to obtain disease-related genes from GeneCards (www.genecards.org), while ‘ferroptosis’ was used to retrieve genes associated with this specific form of programmed cell death.

### Prediction of target genes associated with naringenin, RILI, and ferroptosis

To identify the shared target genes associated with naringenin, RILI, and ferroptosis (Wang X, Meng L, et al. 2023), the research analyzed the intersection of naringenin target genes with RILI and ferroptosis-related genes. A Venn diagram was used to visualize and identify these common target genes. A target network was constructed with Cytoscape, and a protein-protein interaction (PPI) network linking naringenin, RILI, and ferroptosis was built using STRING. These genes can be considered potential targets of naringenin in the treatment of RILI through the ferroptosis pathway.

### Molecular docking of potential targets

The crystal structure of the candidate protein was obtained from the RCSB Protein Data Bank (RCSB PDB) database (www.rcsb.org)(Jiménez-García et al. 2021). Using PyMOL software, ligand and water molecules were removed. Docking parameters were then set, molecular docking was performed using the Swissdock tool, and the results were visualized with UCSF Chimera 1.14 software.

### Cell viability assay

The cells were seeded in 96-well plates (1 × 10^5^/mL). After 24 h for attachment, cells were treated with different concentrations of naringenin (0, 25, 50, 100, and 200 μM) for 24 h. Thereafter, 10 μL of CCK8 (MTT, Sigma-Aldrich, CC) solution was added to each well for 2 h incubation. Finally, absorbance at 450 nm was recorded using a microplate reader.

### Cell culture and treatment

Human normal lung epithelial cells (BEAS-2B) were obtained from Beyotime (C6106, Shanghai, China) and human umbilical vein endothelial cells (HUVECs) were obtained from Procell (CL-0675, Wuhan, China). The cells were cultured in Dulbecco’s Modified Eagle Medium (DMEM) supplemented with 10% fetal bovine serum and 1% penicillin/streptomycin. The DMEM was purchased from WISENT (319-005-CL), and it contained 4.5 g/L D-Glucose, L-Glutamine, phenol red and sodium pyruvate. The cell culture conditions included a stable temperature of 37 °C, a stable CO2 concentration of 5%, and a high relative humidity of 95% saturation. Cells were passaged daily *via* trypsinization. And the third-generation cells were used for experiments. The cells were re-seeded into the well plate. One hour before irradiation, the cell culture medium was removed, and the cells were washed with PBS. Subsequently, cells were pretreated with medium containing naringenin (100 μM) (Zhang et al. 2022). Then, the cells were irradiated with a single dose of 8 Gy using an X-ray linear accelerator (VitalBeam, 4420), and were collected 24 h after irradiation. The whole irradiation process was carried out without replacing the medium, and the medium still contained naringenin. Naringenin (99.27% purity) was purchased from MedChemExpress (Suzhou, China).

### Quantitative real-time PCR (qRT-PCR)

Total RNA was extracted from the cells using Trizol reagent (Vazyme, Nanjing, China) following the manufacturer’s instructions. cDNA was synthesized from the total RNA using the reverse transcription system (YEASEN, Shanghai, China). Quantitative PCR was conducted with SYBR Green (Vazyme, Nanjing, China) using the LightCycler^®^480 detection PCR system (Roche, Foster City, CA). Method 2 ^−ΔΔ^*^CT^* was employed for analysis. All primer sequences are listed in Table 1.

**Table 1.** Primer sequences.

Gene	Forward primer (5′– >3′)	Reverse primer (5′– >3′)
GAPDH	GGAGCGAGATCCCTCCAAAAT	GGCTGTTGTCATACTTCTCATGG
ABCC1	CTCTATCTCTCCCGACATGACC	AGCAGACGATCCACAGCAAAA
AURKA	GAGGTCCAAAACGTGTTCTCG	ACAGGATGAGGTACACTGGTTG
ALOX12	ATGGCCCTCAAACGTGTTTAC	GCACTGGCGAACCTTCTCA
CTSB	GAGCTGGTCAACTATGTCAACA	GCTCATGTCCACGTTGTAGAAGT
ESR1	CCCACTCAACAGCGTGTCTC	CGTCGATTATCTGAATTTGGCCT
FASN	AAGGACCTGTCTAGGTTTGATGC	TGGCTTCATAGGTGACTTCCA
FGFR1	GGCCTATATCCACATCCACAGT	TACCAAGTCCCAGAATGGTGA
GSK3B	GGCAGCATGAAAGTTAGCAGA	GGCGACCAGTTCTCCTGAATC
HNF4A	CACGGGCAAACACTACGGT	TTGACCTTCGAGTGCTGATCC
MAPK1	TACACCAACCTCTCGTACATCG	CATGTCTGAAGCGCAGTAAGATT
MMP2	CCCACTGCGGTTTTCTCGAAT	CAAAGGGGTATCCATCGCCAT
NOX4	CAGATGTTGGGGCTAGGATTG	GAGTGTTCGGCACATGGGTA
PTGS2	CTGGCGCTCAGCCATACAG	CGCACTTATACTGGTCAAATCCC
VCP	CAAACAGAAGAACCGTCCCAA	TCACCTCGGAACAACTGCAAT
IL-1β	TGGTAGTAGCAACCAACGGGA	ACTTTGATTGAGGGCGTCATTC
IL-6	ACTCACCTCTTCAGAACGAATTG	CCATCTTTGGAAGGTTCAGGTTG
TNF-α	CCTCTCTCTAATCAGCCCTCTG	GAGGACCTGGGAGTAGATGAG
GPX4	ATGGTTAACCTGGACAAGTACC	ATGGTTAACCTGGACAAGTACC
FTL	CAGCCTGGTCAATTTGTACCT	GCCAATTCGCGGAAGAAGTG
FTH1	GCTGGTAGAGGAGTGTGCTTGC	GTCCTGGTGGTAGTTCTGGC
SLC7A11	GGCTCCATGAACGGTGGTGTG	GCTGGTAGAGGAGTGTGCTTGC

### Western blot analysis

Western blotting was used to detect levels of Prostaglandin Endoperoxide Synthase (PTGS2), Valosin-containing protein (VCP), and glyceraldehyde-3-phosphate dehydrogenase (GAPDH). Cells were washed twice with cold PBS, and total protein was extracted using RIPA lysis buffer (New Semitech, Suzhou, China). Protein concentration was measured using the BCA protein assay. The proteins were then separated by 10% SDS-PAGE and transferred to a PVDF membrane. The membrane was blocked with 5% skim milk powder for 2 h, incubated overnight at 4 °C with the primary antibody, and then with the secondary antibody for 1–2 h. Band visualization was performed using the Tanon 5200 imaging system. Each experiment was conducted in triplicate. The antibodies used were PTGS2 (12375-1-AP, 1:1000), VCP (10736-1-AP, 1:1000), and GAPDH (10494-1-AP, 1:5000), all purchased from Proteintech (Wuhan, China).

### Immunofluorescence staining assay

The cells were cultured in 12-well plates, fixed with 4% paraformaldehyde (Servicebio), and permeabilized with 0.5% Triton X-100 (Solarbio, Beijing, China). They were then sealed at room temperature with 5% BSA (Solarbio) for 1 h. The cells were incubated overnight at 4 °C with PTGS2 antibody (1:400, Proteintech) and VCP antibody (1:200, Proteintech). Following this, they were incubated with an allogenic secondary antibody (1:200, YEASEN) at room temperature, protected from light, for 1 h. Finally, the nuclei were stained with DAPI and imaged using a Zeiss microscope.

### Intracellular ROS level detection

The ROS levels in cells were measured using the ROS detection kit (Nanjing Jian Cheng, Nanjing, China). Cells pretreated with naringenin for 1 h were incubated at 37 °C for 24 h post-irradiation. They were then washed with PBS. The ROS probe was diluted 1:1000 in serum-free culture medium, and 1 mL of this diluted probe was added to each well of a 6-well plate. The cells were incubated at 37 °C in the dark for 30 min. Following this, the cells were washed with PBS three times, and 1 mL of medium was added to observe ROS production.

### Statistical analyses

All statistical analyses were conducted using GraphPad Prism 9.0, and data are presented as mean ± standard deviation. Behavioral data were analyzed using repeated measures ANOVA, while other data were compared between groups using one-way ANOVA followed by Tukey’s multiple comparisons test. A *p*-value of <0.05 was considered statistically significant.

## Results

### Pharmacokinetic parameters of naringenin

**Figure 2.**
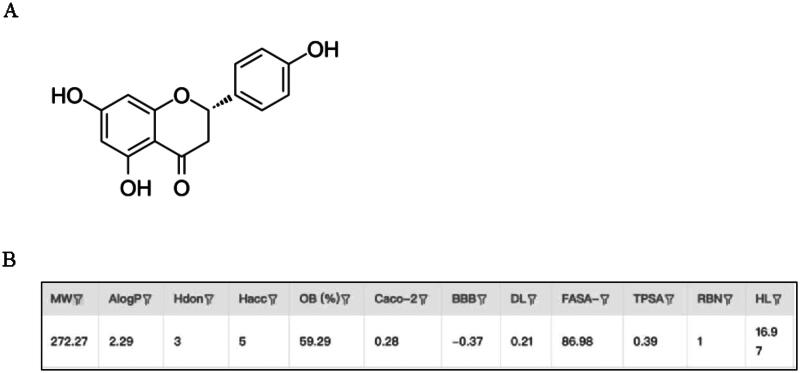
Naringenin relevance. (A) 2D molecular structure of naringenin. (B) Pharmacokinetic parameters of naringenin comeing from the TCMSP.

The pharmacokinetic parameters of naringenin were obtained from the TCMSP database. The molecular ID for naringenin is MOL004328, with a molecular formula of C_15_H_12_O_5_ and a molecular weight (MW) of 272.27 (Figure 2(A)). The lipophilicity partition coefficient (AlogP) is 2.29, indicating the compound’s lipophilic nature (Wardecki et al. 2023), while the intestinal epithelial permeability (Caco-2) value is 0.28, suggesting moderate absorption through the intestine. Naringenin has an oral bioavailability (OB) of 59.29% (Panse and Gerk 2022), wherein an OB above 30% is generally considered therapeutically effective. The blood-brain barrier permeability (BBB) value is −0.37, indicating poor penetration of the blood-brain barrier. Additionally, naringenin has a half-life (HL) of 16.97 h and a drug-likeness (DL) value of 0.21, surpassing the common threshold value of 0.18 for potential drug consideration (Figure 2(B)). These parameters collectively suggest that naringenin has potential as a therapeutic drug with moderate water solubility, good oral availability, and promising development prospects. Naringenin is a dihydroflavonoid. It composed of 3 hydroxyl functional groups and 1 ketocarbonyl group, in which the hydroxyl groups are crucial in determining the physiological activity of naringenin to scavenge free radicals, thereby exerting an antioxidant effect that mitigates oxidative stress and, consequently, helps to reduce inflammation (Singh et al. 2023).

### The naringenin effects of anti-inflammatory and inhibitory ferroptosis in radiation-induced cell injury

**Figure 3.**
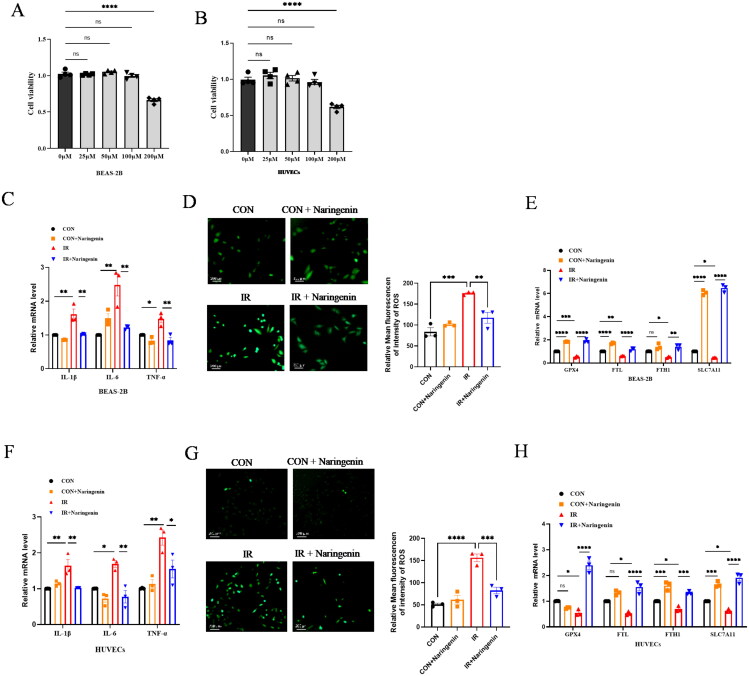
The effect of naringenin in RILI. (A) Naringenin of CCK-8 assay analysis in BEAS-2B and (B) HUVECs. (C) Evaluation of the mRNA levels of inflammatory factor (IL1-β, IL-6, TNF-α) using RT-PCR in BEAS-2B.(D) the ROS intensity detection of BEAS-2B. (E) the mRNA levels of ferroptosis related factor (GPX4, FTL, FTH1, SLC7A11) in BEAS-2B. (F) The mRNA levels of inflammatory factor (IL1-β, IL-6, TNF-α) in HUVECs. (G) The ROS intensity detection of HUVECs. (H) the mRNA levels of ferroptosis related factor (GPX4, FTL, FTH1, SLC7A11) in HUVECs cell. *p < 0.05, **p < 0.01, ***p < 0.001 and ^****^p < 0.0001.

The cells, BEAS-2B and HUVECs, treated with or without naringenin 1 h before exposure to 8 Gy irradiation were divided into four groups: control group, naringenin group, irradiation group, and irradiation-naringenin group. Cell viability was determined using a CCK-8 assay after treatment with naringenin at concentrations of 0, 25, 50, 100, and 200 μM (Figure 3(A,B))(Zhang et al. 2022). Ultimately, the cells were pre-treated with 100 μM naringenin and collected 24 h after irradiation. To understand the effects of naringenin on radiation-induced cell damage, inflammatory cytokine mRNA levels and ROS levels were examined. The mRNA levels of inflammatory cytokines IL-1β, IL-6, and TNF-α in BEAS-2B and HUVECs were significantly increased after irradiation but were significantly decreased with naringenin treatment (Figure 3(C,F)). Additionally, the fluorescence intensity of ROS in the irradiation group was significantly higher than in the control group, whereas it was significantly decreased with naringenin treatment (Figure 3(D,G)). These findings suggest that naringenin has potent anti-inflammatory effects and inhibits ROS production in epithelial and endothelial cells. Meanwhile, this study found that naringenin could correct the imbalance of ferroptosis-related genes expression levels in radiation-induced cell damage (Figure 3(E,H)).

### Construction and analysis of the naringenin-RILI-ferroptosis target genes network

**Figure 4.**
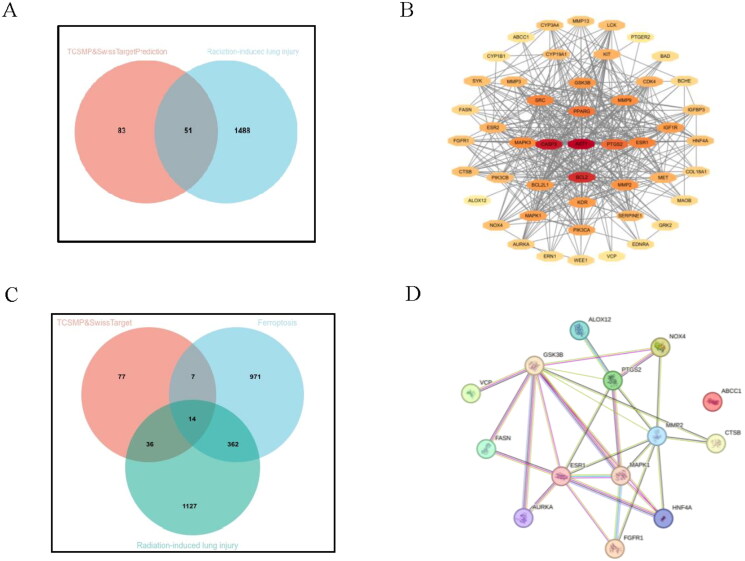
Potential targets of naringenin in the context of RILI and ferroptosis. (A) A Venn diagram illustrating the common targets associated with naringenin and RILI. (B) The target network of the Naringenin-RILI common genes constructed using Cytoscape. (C) A Venn diagram summarizing the 14 key genes common targets associated with naringenin and RILI in the ferroptosis. (D)The protein–protein interaction (PPI) network of the ‘Naringenin-RILI-Ferroptosis’ common genes constructed using STRING. RILI: radiation lung injury.

This experiment retrieved 37 targets related to naringenin from the TCMSP database and 100 targets from the SwissTargetPrediction database, with a total of 3 common targets identified in both databases. By consolidating these common targets, combining similar entries, and removing duplicates, a total of 134 unique targets were obtained. Additionally, the GeneCards database provided 1539 genes associated with RILI and 1,354 genes linked to ferroptosis. Mapping the naringenin-related targets to RILI genes resulted in 51 intersecting drug-disease genes (Figure 4(A)). These 51 shared targets were imported into Cytoscape to construct the ‘drug-disease target’ interaction network, with 3 targets not related to potential action being removed (Figure 4(B)). To further explore potential targets involving naringenin, RILI, and ferroptosis, these three sets of target genes were combined, and 14 overlapping targets were identified for naringenin’s role in treating RILI through ferroptosis (Figure 4(C)). Using the STRING website, these 14 genes were mapped into a PPI network (Figure 4(D)).

### Determination of the key target genes: PTGS2 and VCP

**Figure 5.**
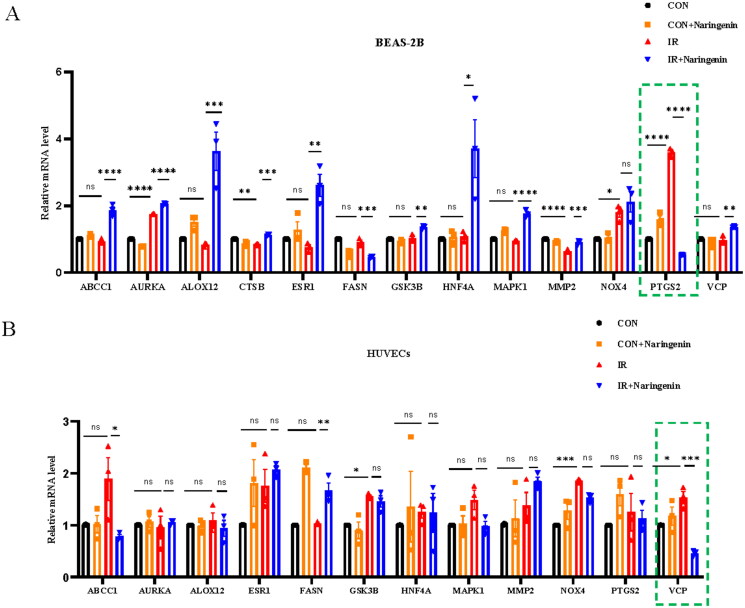
The mRNA levels. (A) Evaluation of the mRNA levels of 14 common target genes in BEAS-2B and (B) HUVECs. *p < 0.05, **p < 0.01, ***p < 0.001 and ^****^p < 0.0001.

The mRNA levels of 14 target genes in the PPI network confirmed PTGS2 and VCP as key target genes (Neither cell lines expressed FGFR1, and HUVECs specifically did not express CTSB) (Figure 5(A,B)). Significant changes were observed in AURKA, CTSB, MMP2, and PTGS2 in BEAS-2B cells after irradiation (Figure 5(A)). Notably, mRNA levels of PTGS2, a recognized gene associated with ferroptosis, significantly increased in the irradiated group compared to the control group (*p* < 0.0001), but significantly decreased after treatment with naringenin (*p* < 0.0001). In HUVECs, significant changes were observed only in VCP after irradiation (*p* < 0.05). VCP mRNA levels significantly increased after irradiation and significantly decreased after naringenin treatment (*p* < 0.001) (Figure 5(B)).

### Molecular docking verification

**Figure 6.**
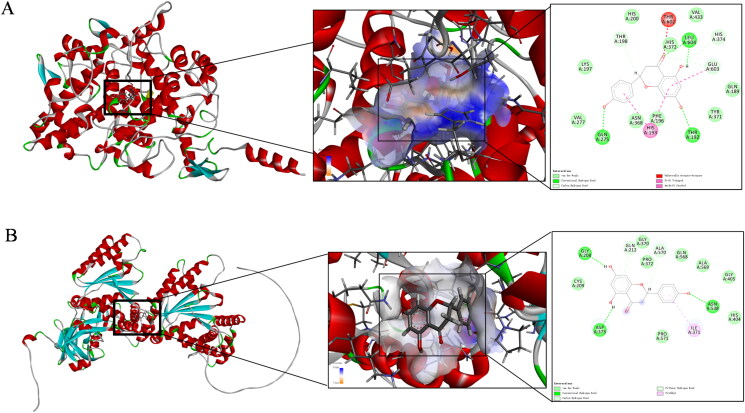
Molecular docking prediction. (A) Molecular docking of naringenin with PTGS2 protein. (B) Molecular docking of naringenin with VCP protein.

The docking of naringenin with the top hub proteins, PTGS2 and VCP, were shown in Figure 6. The main interactions between naringenin and the residues within the PTGS2 and VCP binding pockets were analyzed. Naringenin interacts with the residues THRA: 602, LEUA: 604, THRA: 192, GLNA: 275, and HISA: 193 of PTGS2 to maintain structural stability. This was followed by the formation of unfavorable acceptor-acceptor interactions, conventional hydrogen bonds, Pi-Pi T-shaped bonds, and van der Waals forces between naringenin and PTGS2 (Figure 6(A)). For VCP, naringenin interacts with the residues GLYA: 208, ASPA: 373, ASNA: 538, and ILEA: 371 to maintain structural stability, forming Pi-donor hydrogen bonds, Pi-alkyl bonds, and van der Waals forces (Figure 6(B)).

### *Validation of PTGS2 and VCP genes* in vitro

**Figure 7.**
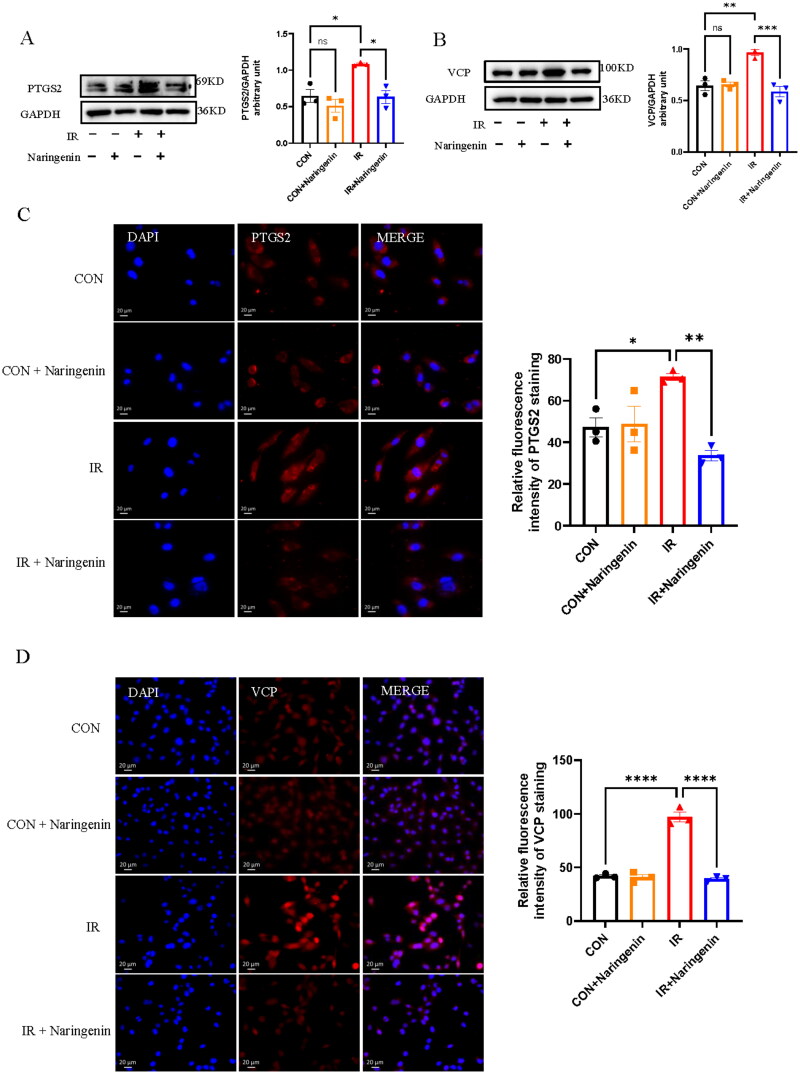
Western blot analysis and immunofluorescence of PTGS2 and VCP. (A) Protein expression levels of PTGS2 were assessed in irradiation induced BEAS-2B treated with naringenin. (B) Protein expression levels of VCP were assessed in HUVECs. (C) Immunofluorescence staining was employed to detect the expression of PTGS2 in BEAS-2B, (D) and VCP in HUVECs. *p < 0.05, **p < 0.01, ***p < 0.001 and ^****^p < 0.0001.

The changes of PTGS2 and VCP expression in the RILI model after naringenin treatment were further verified by Western blot analysis. PTGS2 levels significantly increased in BEAS-2B epithelial cells after irradiation and significantly decreased following naringenin treatment (Figure 7(A)). In endothelial cells (HUVECs), VCP levels increased after irradiation and decreased after naringenin treatment (Figure 7(B)). Immunofluorescence analysis of the cells confirmed these findings (Figure 7(C,D))

## Discussion

In this study, naringenin was found to ameliorate the radiation-induced damage to BEAS-2B and HUVECs cells by inhibiting ferroptosis. Furthermore, PTGS2 and VCP were identified as novel key genes that exacerbate damage to both cells *via* ferroptosis.

RILI is a common clinical complication in thoracic tumor patients undergoing radiotherapy (Giuranno et al. 2019). However, there is no effective treatment for RILI, and its potential mechanism remains unclear (Xia et al. 2020). Highly inflammatory cytokines (IL-1β, IL-6, and TNF-α) are generally considered as markers of RILI, while ROS are the key factors in RILI (Szabo et al. 2010; Wang L et al. 2023). Naringenin, a potent active compound, has gained significant attention due to its notable antioxidant, anti-inflammatory, and anticancer properties (Joshi et al. 2018; Motallebi et al. 2022). Naringenin possesses a remarkable ability to reduce inflammation across a wide range of diseases (Wang et al. 2020; Wang Y et al. 2023), which is consistent with the experimental results that naringenin can significantly reduce the levels of ROS and pro-inflammatory cytokines (IL-1β, IL-6 and TNF-α). Therefore, naringenin, as a powerful anti-inflammatory and antioxidant compound, is expected to become a powerful and effective drug for the treatment of RILI.

Ferroptosis is a newly recognized form of cell death that plays a potential physiological role in a variety of diseases (Jiang et al. 2021; Cao et al. 2022). Inhibition of ferroptosis has been shown to alleviate pathological changes in acute RILI (Li et al. 2022). Previous studies showed that naringenin exhibits the ability to alleviate myocardial ischemia/reperfusion injury, silver nanoparticle-induced lung injury, and cigarette-induced lung injury by inhibiting ferroptosis (Zhang et al. 2022, 2023; Fan et al. 2024). In this study, naringenin was found to reduce the mRNA levels of ferroptosis-related genes including *GPX4, FTL, FTH1*, and *SLC7A11* in radiation-induced cell damage. Although the relationship between naringenin, ferroptosis, and RILI has not been studied extensively, based on previous studies and the results of this study, it can be concluded that naringenin alleviates radiation-induced cell damage by inhibiting ferroptosis.

In addition, computer simulation analysis was performed to explore the potential targets of naringenin in inhibiting ferroptosis and alleviating radiation-induced cell damage. Consequently, PTGS2 and VCP were identified as key genes. Notably, after radiation exposure, the expression levels of PTGS2 and VCP were significantly upregulated in BEAS-2B cells and HUVECs, respectively. Naringenin exhibited the ability to reverse this alteration. The inconsistencies between PTGS2 and VCP in the epithelium and endothelium may be due to the different functions of the two cells in RILI (Guo et al. 2024). This study demonstrated that PTGS2 and VCP play important roles in naringenin alleviating radiation-induced cell damage through ferroptosis. Additionally, the significance of PTGS2 has also been identified in various other diseases. Previous studies also supported that PTGS2 is considered as a key gene in influencing the prognosis of cervical cancer (Zou et al. 2022), and its protein product function as a potential therapeutic target for atherosclerosis since PTGS2 served as a hub gene in regulatory networks (Zhou et al. 2021). Therefore, PTGS2 and VCP may also be served as predictive indicators for the occurrence of RILI. Furthermore, the therapeutic effect of naringenin on RILI may be involved in restraining the expression of PTGS2 and VCP, thereby inhibiting ferroptosis and alleviating RILI.

However, there were certain restrictions in this study. Our study was conducted only *in vitro* but was not validated *in vivo*. In addition, our research offered preliminary evidence of the PTGS2 and VCP as potential targets of naringenin by inhibiting ferroptosis in treating RILI, but it did not fully explore the molecular mechanism, the formation of RILI may involve more regulatory mechanisms.

## Conclusions

The study offers new insights into RILI by identifying PTGS2 and VCP as key target genes through which naringenin acts to counteract radiation-induced cell damage *via* ferroptosis inhibition. In the future, building upon the current research findings, we aim to further investigate the pathways and interactions between ferroptosis-related genes (PTGS2, VCP) and RILI, explore novel mechanisms linking ferroptosis and RILI, and devise potential new therapeutic strategies to manage RILI.

## Data Availability

The data that support the finding of this study are openly available in TCMSP (https://tcmspw.com/tcmsp.php)(Ru et al. 2014), GeneCards (https://wwwgenecards.org)(Stelzer et al. 2016), Venn (https://bioinfogp.cnb.csic.es/tools/venny/z)(Jia et al. 2021), UniProt (https://www.uniprot.org/)(Hinz 2010), String (https://string-db.org/), SwissTargetPrediction (http://swisstargetprediction.ch)(Daina et al. 2019), RCSB Protein Data Bank (https://www.rcsb.org) (Berman et al. 2000), and the analysis software used in this study include Cytoscape 3.7.1, and PyMOL (https://pymol.org/2) (Del Conte et al. 2023).
